# Evaluation of stool microbiota signatures in two cohorts of Asian (Singapore and Indonesia) newborns at risk of atopy

**DOI:** 10.1186/1471-2180-11-193

**Published:** 2011-08-26

**Authors:** Gaik Chin Yap, Kok Keong Chee, Pei-Ying Hong, Christophe Lay, Cahya D Satria, Sumadiono, Yati Soenarto, Ekawaty L Haksari, Marion Aw, Lynette Pei-Chi Shek, Kaw Yan Chua, Yudong Zhao, Doreen Leow, Bee Wah Lee

**Affiliations:** 1 Department of Paediatrics, National University of Singapore, Medical Drive, Singapore, 117597 Singapore; 2 Department of Animal Sciences, University of Illinois, West Gregory Drive,Urbana-Champaign, 61801, USA; 3 Genome Institute of Singapore, Biopolis Street, Singapore, 138672, Singapore; 4 Faculty of Medicine, Gadjah Mada University, Jalan Kesehatan, Yogyakarta, 55284 Indonesia; 5 Singapore Clinical Research Institute, Biopolis Way, Singapore, 138669, Singapore

## Abstract

**Background:**

Studies have suggested that demographic and lifestyle factors could shape the composition of fecal microbiota in early life. This study evaluated infant stool microbiota signatures in two Asian populations, Singapore (n = 42) and Indonesia (n = 32) with contrasting socioeconomic development, and examined the putative influences of demographic factors on these human fecal associated bacterial signatures.

**Results:**

Longitudinal analysis showed associations of geographical origin with *Clostridium leptum, Atopobium *and *Bifidobacterium *groups. Mode of delivery had the largest effect on stool microbiota signatures influencing the abundance of four bacterial groups. Significantly higher abundance of bacterial members belonging to the *Bacteroides-Prevotella, Bifidobacterium *and *Atopobium *groups, but lower abundance of *Lactobacilli-Enterococci *group members, were observed in vaginal delivered compared to caesarean delivered infants. Demographic factors influencing the structure of infants stool microbiota during the first year of life included breastfeeding, age of weaning, sibship size and exposure to antibiotics.

**Conclusions:**

Differences in stool microbiota signatures were observed in relation to various demographic factors. These features may confound studies relating to the association of the structure of fecal microbiota and the predisposition to human modern disease.

## Background

The human gut microbiome is a complex ecosystem harbouring a rich diversity of commensal microorganisms. It is widely thought that the early life development of the neonatal intestinal microbiota plays an important role in the maturation of the host immune system and could in turn influence allergy development [1-3]. For example, germfree mice which lack the endemic intestinal microbiota showed impairment of intestinal mucosal and systemic immune system development. The impairment in the systemic immune system is reflected by poorly formed spleen and lymph nodes, hypoplastic Peyer's patches, reduced levels of secreted IgA and IgG, and lack of expansion of CD4+ T cell populations [2,3]. Furthermore, these mice exhibited cytokine profiles that skewed towards Th2 [2], which is involved in the pathophysiology of allergic diseases.

Past studies have further reported that intestinal microbiota in subjects with allergy, particularly those with atopic eczema, differed from those of healthy controls [4-7]. Wang and colleagues showed that there is a reduced bacterial diversity in the early stool microbiota of infants with atopic eczema [7]. Recently, we further showed that the abundances of *Bifidobacterium *and Enterobacteriaceae were different among caesarean-delivered infants with and without eczema [5]. The association with specific gut microbiota signatures and the occurrence of allergy in infants has also been reported in studies comparing healthy and allergic infants from different countries [8,9].

However, varying demographic and lifestyle characteristics at different geographical locations can pose as potential confounders in correlating multidimensional data generated from studies involving the diverse bacterial populations of the gut microbiota as "quantitative traits". For example, factors that have been shown to influence gut microbiota colonization in early life include the mode of delivery of the newborn, infant feeding pattern, and household factors such as sibship size [8,10-12]. Additionally, medication such as the use of antibiotics may also influence the pattern of intestinal microbiota colonization [10,11]. Across geographical locations, socioeconomic and cultural differences would result in a significant variance in the mothers' choice of dietary regimen for their infants, the number of children born within a household (i.e., sibship size) and so on. Therefore, prior to examining the correlation between host health status and gut microbiota, it is essential to better elucidate how the gut microbiota would be affected by the various demographic and lifestyle factors arising from living in different geographic locations.

Our study aimed to investigate the influence of demographic factors on determining the microbial colonization of the infant colon in two Asian populations, Singapore (SG) and Yogyakarta, Indonesia (IN). SG represents an affluent and urbanized community, and IN being an urbanized but developing community. We employed molecular techniques: terminal restriction fragment length polymorphism (T-RFLP) and fluorescent *in situ *hybridization combined with flow cytometry (FISH-FC) targeting seven major bacterial groups to evaluate and monitor the structure of the colonic microbiota at four time points (i.e, 3 days, one month, three months and one year of age). This study would provide insight on the infant gut microbial succession pattern, as well as the demographic factors that influence stool microbiota signatures in these two Asian populations over the first year of life.

## Results

### Demographic and Clinical Characteristics

The demographic and clinical characteristics are shown in both Singaporean (SG) and Indonesian (IN) populations (Table 1). Vaginal delivery was more common in SG compared to IN (p = 0.019). In early infancy till 6 months, 85.7% and 80.7% of the SG and IN cohorts, respectively, opted for partial breast and formula feeding. There were a higher percentage of Indonesian infants who were exclusively breastfed in the first 6 months (18.72%, 6/32). In contrast, none of the Singaporean infants were exclusively breastfed for that period of time (p = 0.004). Instead, more SG infants (14.3%) were exclusively formula fed in the first 6 months compared to none in the IN cohort (p = 0.035). Weaning to semisolids for IN cohort occurred later than SG cohort (6.72 months versus 3 months, respectively; p = 0.022). Prenatal antibiotics were administered only in IN cohort (p = 0.013), but the number of infants from both cohorts who received postnatal antibiotics were not significantly different. No significant differences arising from the geographic locations were observed for factors such as gender proportion, postnatal antibiotics consumption and sibling number.

**Table 1:** Demographic characteristics of Singapore (n = 42) and Indonesia (n = 32) children

	Indonesia (n = 32)	Singapore (n = 42)	*p *value
**Gender (%)**			
Male	22 (68.75)	24 (57.1)	0.308
Female	10 (31.25)	18 (42.9)	
			
**Mode of Delivery (%)**			
Vaginal delivery	16 (50)	32 (76.2)	0.019*
Lower Segment caesarean section	16 (50)	10 (23.8)	
			
**Feeding history from birth to month 6 (%)**			
Total breastfeeding	6 (18.75)	0 (0)	0.005*
Breastfeeding and formula feeding	26 (81.25)	36 (85.71)	0.606
Total formula feeding	0(0)	6 (14.29)	0.033*
			
**Eczema (%)**			
Yes	6 (18.75)	13 (31)	0.234
			
**Antibiotics (%)**			
Prenatal (Yes)	5 (15.6)	0 (0)	0.013*
Postnatal (Yes)	8(25.0)	16 (38.1)	0.233
			
**Age at weaning (months)**			
Mean (SD)	6.73 (1.892)	5.63 (0.773)	0.007*
Median (Range)	6 (3-11)	6 (4-7)	
			
**Number of siblings**			
Mean (SD)	0.78 (1.039)	1.24 (1.34)	0.113
Median (Range)	0 (0-4)	1 (0-6)	
			

* Statistically significant differences are indicated (p < 0.05)

### Temporal change of relative abundance of seven bacterial groups

**Figure 1:**
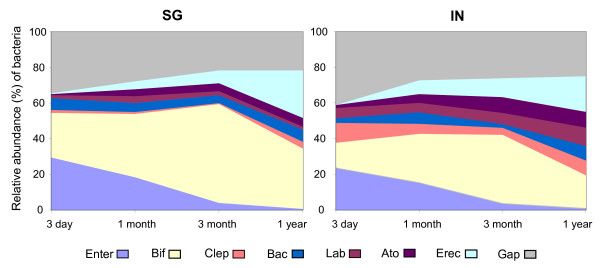
**Comparison of relative abundance of seven predominant bacterial groups between Singapore and Indonesia infants**. Singapore cohort is represented by SG while Indonesia cohort is represented by IN. Enterobacteriaceae (Enter), *Bifidobacterium *(Bif), *Clostridium leptum *(Clep), *Bacteroides-Prevotella *(Bac), *Lactobacilli-Enterococci *(Lab), *Atopobium *(Ato), *Eubacterium rectale-Clostridium coccoides *(Erec), and phylogenetic gap (Gap) represent the remaining microbiota that was not targeted by our panel of probes.

The relative abundance of seven bacterial groups was quantified (Figure 1). Although the proportions differed, the trends of bacterial colonization studied over the first year of life were similar for SG and IN cohorts (Figure 1). For example, in both SG and IN cohorts, members of the Enterobacteriaceae family, were one of the earliest colonizers and gradually decreased to an average 0.67% of total bacteria counts at 1 year of age. Colonization of *Eubacterium rectale-Clostridium coccoides *group increased gradually from 0.18% to 24.07% of total bacteria at 1 year old. The colonization pattern of *Bifidobacterium *showed an initial increase from a mean of 19.92% at 3 days to 49.50% at 3 months but later decreased to 27.34% at one year of age. A reversal of pattern was seen with *Clostridium leptum *group where a decrease in colonization from a mean of 5.88% to 1.59% occurred between 3 days and 3 months of age but increased subsequently at the age of one year. The other three bacterial groups such as *Bacteroides-Prevotella, Atopobium *and *Lactobacilli-Enterococci *group remained in relatively lower abundance throughout the first year of life, and each constituted less than 10% of the total bacteria detected in stool sample throughout all time points. The phylogenetic gap included the remaining bacterial members that were not targeted by our panel of probes, and the relative abundance of the phylogenetic gap ranged from 22.89% to 37.40% of total bacteria.

### Influence of Demographic and Lifestyle/Clinical Characteristics

We comparatively examined the abundances of bacterial groups in relation to demographic factors: geographical origin, mode of delivery, dietary regimen and weaning age, and sibship size. These demographic characteristics differed between the SG and IN cohorts (Table 1), and we aimed to determine if these demographic factors would result in corresponding differences in the abundance of specific fecal associated bacterial groups. The additional file 1 details the univariate analysis of relative abundance (%) of seven bacterial group members of the infant fecal microbiota in relation to geographical and clinical factors.

#### (A) Geographical Origin

**Figure 2:**
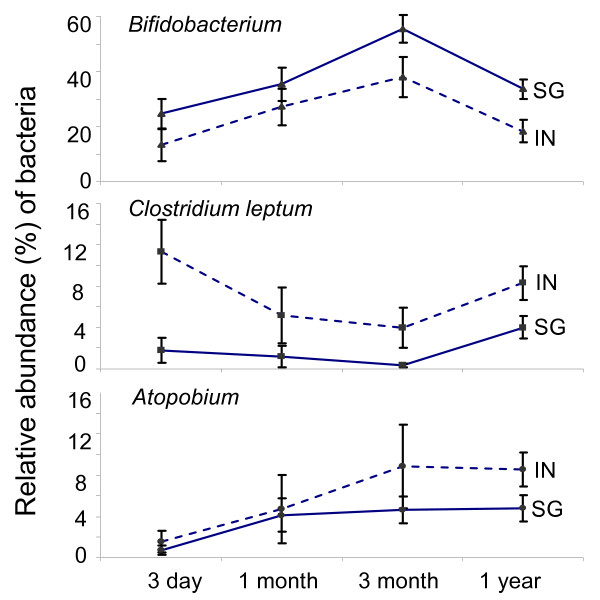
**Longitudinal comparison of fecal microbiota by geographical origin of subjects**. Linear mixed model analysis involved adjustment against other confounding factors (Mode of delivery, weaning age, sibling number, total breastfeeding up to 6 month, eczema and prenatal antibiotics). Indonesia cohort (IN) represented by dotted line, and Singapore cohort (SG) represented by solid line.

Three bacterial groups, namely *Clostridium leptum, Atopobium *and *Bifidobacterium*, differed in abundance between the SG and IN cohorts. Linear mixed model revealed that the relative abundances of *Clostridium leptum *[coefficient (B): 7.758, 95% confidence interval (CI): 5.063-10.453, adj p < 0.001] and *Atopobium *group [B: 3.526, 95%CI: 1.102 - 5.949, adj p = 0.005] were higher in IN cohort (Figure 2). Conversely, lower relative abundance of *Bifidobacterium *was observed in the IN cohort [B: -13.950, 95%CI: -26.423 - -1.476), adj p = 0.029] (Figure 2).

#### (B) Mode of Delivery

**Figure 3:**
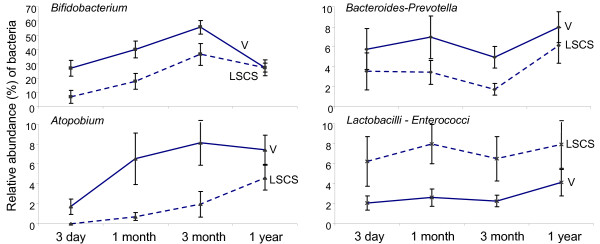
**Longitudinal comparison of fecal microbiota by mode of delivery**. Linear mixed model analysis adjusted with confounding factors (Location, weaning age, sibling number, total breastfeeding up to 6 month, eczema and prenatal antibiotics). Caesarean delivery (LSCS) represented by dotted line, and vaginal delivery (V) represented by solid line.

Longitudinal analyses over four time points showed that mode of delivery had the largest effect on the abundance of four bacterial groups (Figure 3). In vaginal delivered infants, significantly higher abundance of *Bacteroides-Prevotella *[B: 3.016, 95%CI: 0.639 - 5.394, adj p = 0.014], *Bifidobacterium *[B: 16.040, 95%CI: 5.667 - 26.414, adj p = 0.003] and *Atopobium *group [B: 2.531, 95%CI: 0.472 -4.589, adj p = 0.017] were observed. However, lower abundance of *Lactobacilli - Enterococci *group [B: -3.665, 95%CI: -5.949 - -1.381, adj p = 0.002] was observed in vaginal delivered infants.

#### (C) Breastfeeding and Age of Weaning

The relative abundance of *Lactobacilli-Enterococci *group was significantly higher in infants who were exclusively breastfed throughout all time points [B: 5.236, 95%CI: 1.044 - 9.428, adj p = 0.015] (Table 2). Another factor that affected the abundance of stool microbiota was the age of weaning to semisolids. Linear mixed model showed a decrease in abundance of *Clostridium leptum *group for every month of increase in weaning age [B: -0.827, 95%CI: -1.5934 - -0.0602, adj p = 0.035].

**Table 2:** Feeding habits and demographic factors affecting the relative abundance of microbial groups

	Bacteria groups	Mean differences (95% CI)	*p *value
Total breastfeeding: Yes versus No	*Lactobacilli - Enterococci*	5.236 (1.044 - 9.428)	0.015
Weaning age	*Clostridium leptum*	-0.827 (-1.5934 - -0.0602)	0.035
Sibling number	*Bifidobacterium* *Enterobacteriaceae*	3.873 (0.112 -7.634) -0.526 (-0.8725 - -0.1801)	0.044 0.004

Linear mixed model analysis adjusted with confounding factors (Location, mode of delivery, weaning age, sibling number, total breastfeeding up to 6 month, eczema and prenatal antibiotics). Only bacteria groups with statistically significant differences are listed.

#### (D) Sibship Size

Relative abundance of *Bifidobacterium *increased by 3.873% with every increase in sibling number [B: 3.873, 95%CI: 0.112 -7.634, adj p = 0.044]. On the other hand, the abundance of Enterobacteriaceae decreased with each increase in sibship size [B: -0.526, 95%CI: -0.8725 - -0.1801, adj p = 0.004] (Table 2).

#### (E) Exposure to Antibiotics

The relative abundance of *Clostridium leptum *group at 1 year of age was significantly higher for infants that reported their postnatal antibiotic intake at period of 6 months to 1 year of age [B: 5.706; adj p = 0.025], as compared to the infants who did not consume antibiotics.

### Stool Microbial Richness/Diversity

T-RFs of stool microbiota in SG and IN cohorts were obtained from three individual enzymatic digestions (i.e., AluI, MspI and RsaI), and compared for their microbial richness based on Shannon and Simpson Index. Microbial richness between the cohorts was considered different when both Shannon and Simpson Index from all three enzymatic digestions were significantly different. Table 3 shows that there were no observable differences in the microbial richness of SG and IN cohorts at both 3 months and 12 months of age, both before and after adjusting for demographic confounders. In contrast, when the infants from both geographical locations were grouped according to their mode of delivery, microbial richness of stool microbiota in vaginal-delivered infants had a significantly higher microbial richness compared to caesarean-delivered infants at 12 months of age (Table 3). The microbial richness of stool microbiota did not correlate with other lifestyle factors.

**Table 3:** Shannon and Simpson diversity index determined from T-RFLP profiles

Time	Index of diversity		Location		Mode of delivery	
			Indonesia (n = 19)	Singapore (n = 29)	Vaginal (n = 32)	Caesarean (n = 16)
3 month	Shannon AluI	mean (SD)	1.648 (0.658)*	1.211 (0.643)*	1.350 (0.706)	1.452 (0.635)
		median (range)	1.714 (0.211-2.723)*	1.224 (0-2.371)*	1.424 (0-2.723)	1.415 (0.211-2.647)
	Simpson AluI	mean (SD)	0.685 (0.222)	0.530 (0.261)	0.579 (0.268)	0.617 (0.237)
		median (range)	0.768 (0.085-0.914)	0.568 (0-0.882)	0.667 (0.914)	0.669 (0.085-0.908)
	Shannon MspI	mean (SD)	1.474 (0.647)	1.402 (0.503)	1.408 (0.544)	1.477 (0.605)
		median (range)	1.412 (0.522-2.801)	1.379 (0.228-2.131)	1.378 (0.228-2.672)	1.508 (0.523-2.801)
	Simpson MspI	mean (SD)	0.634 (0.198)	0.627 (0.193)	0.626 (0.190)	0.638 (0.207)
		median (range)	0.652 (0.220-0.916)	0.692 (0.085-0.851)	0.662 (0.085-0.905)	0.697 (0.220-0.916)
	Shannon RsaI	mean (SD)	1.689 (0.597)	1.552 (0.497)	1.621 (0.517)	1.577 (0.591)
		median (range)	1.709 (0.339-2.635)	1.539 (0.643-2.507)	1.664 (0.643-2.514)	1.659 (0.339-2.635)
	Simpson RsaI	mean (SD)	0.711 (0.185)	0.697 (0.177)	0.718 (0.159)	0.671 (0.214)
		median (range)	0.760 (0.162-0.898)	0.737 (0.317-0.979)	0.745 (0.384-0.979)	0.734 (0.162-0.898)
			**Indonesia (n = 29)**	**Singapore (n = 41)**	**Vaginal (n = 46)**	**Caesarean (n = 24)**
1 year	Shannon AluI	mean (SD)	2.102 (0.594)*	1.861 (0.423)*	2.089 (0.409)*	1.715 (0.601)*
		median (range)	2.107 (0.558-2.822)*	1.976 (0.803-2.574)*	2.089 (0.940-2.822)*	1.708 (0.558-2.697)*
	Simpson AluI	mean (SD)	0.785 (0.168)	0.759 (0.120)	0.804 (0.104)*	0.704 (0.179)*
		median (range)	0.837 (0.226-0.925)	0.796 (0.434-0.905)	0.824 (0.434-0.925)*	0.742 (0.226-0.917)*
	Shannon MspI	mean (SD)	1.910 (0.753)*	1.740 (0.430)*	1.992 (0.456)*	1.462 (0.658)*
		median (range)	1.929 (0.252-3.199)*	1.8 (0.777-2.478)*	1.961 (1.137-3.199)*	1.473 (0.252-2.919)*
	Simpson MspI	mean (SD)	0.744 (0.186)	0.747 (0.101)	0.795 (0.086)*	0.650 (0.175)*
		median (range)	0.788 (0.160-0.951)	0.766 (0.462-0.882)	0.806 (0.614-0.951)*	0.686 (0.160-0.935)*
	Shannon RsaI	mean (SD)	2.026 (0.600)	1.965 (0.379)	2.148 (0.334)*	1.688 (0.572)*
		median (range)	2.020 (0.376-2.890)	1.985 (0.874-2.561)	2.181 (1.533-2.890)*	1.765 (0.376-2.868)*
	Simpson RsaI	mean (SD)	0.772 (0.170)	0.797 (0.097)	0.829 (0.064)*	0.706 (0.183)*
		median (range)	0.806 (0.165-0.925)	0.820 (0.459-0.902)	0.846 (0.681-0.925)*	0.776 (0.165-0.925)*

16S rRNA gene amplicons from infant fecal sample were digested with three restriction enzymes (AluI, MspI and RsaI).

*Statistically significant differences were indicated (adjusted p < 0.05). Linear regression for location and mode of delivery were performed with adjustment for confounding factors (Location, mode of delivery, weaning age, sibling number, total breastfeeding up to 6 month, eczema and prenatal antibiotics). Missing data was reported due to lack of availability of DNA samples for certain subjects.

## Discussion

In this study, we first examined the temporal succession of specific bacterial groups in the infant stool microbiota sampled from two geographical locations (i.e., Singapore and Yogyakarta, Indonesia). These two populations in Asia are considered diverse, in terms of geographical distance, cultural ethnicity and overall social economic development. Despite these differences between the two populations, our findings showed that the dynamic of colonization was similar in both cohorts. For example, Enterobacteriaceae and *Bifidobacterium *constitute the predominant bacterial groups in stool microbiota before three months of age, and were present at a relative abundance of up to 98% of total bacteria. This observation is in agreement with past reports which found that healthy infants from Netherlands, breastfed Indian infants from Guatemala, preterm infants from Nigeria and 6-week old infants across Europe also had a similar predominance of Enterobacteriaceae and *Bifidobacterium *[10,13-15]. As the infants age, our study also showed that Firmicutes represented by members of the *Eubacterium rectale-Clostridium coccoides *group increased in its abundance, and gradually resembled that of an adult stool microbiota i.e. mainly populated with members of the Firmicutes and Bacteroidetes phylum [16]. The similarities in the pattern of colonization from early till late infancy despite geographical differences may be related to multiple factors. For example, the prevalence of facultative anaerobes Enterobacteriaceae during early life may be due to a relatively aerobic gastrointestinal tract, and the need for the facultative anaerobes to deplete the oxygen content so as to provide an anoxic environment suitable for other commensal microbes to establish [17].

There remains no clear explanation for the predominance of *Bifidobacterium *in most infants, including those who were exclusively formula-fed, but not in adults. A possible reason may be related to the diet consumed by the human host at different stages of life. To illustrate, dietary carbohydrates that are consumed by infants comprise mainly disaccharides (lactose) and oligosaccharides [18,19], which are in turn rapidly hydrolyzed to form galactose and glucose monosaccharides [20,21]. A portion of these monosaccharides becomes available for the commensal microbiota, and because *Bifidobacterium *spp. produce more ATP per mole of glucose through the bifidus pathway [22], there remains a selective advantage for *Bifidobacterium *to out compete the other commensal bacterial groups fermenting carbohydrates through the conventional glycolysis and 6-phosphogluconate pathways. Subsequently, as the host matures and undergoes weaning, the dietary carbohydrates become more complex and eventually favour the establishment of other bacterial members belonging to the Bacteroides and *Clostridium *for instance, which are known to contain a wide repertoire of polysaccharides-utilizing gene clusters that can effectively degrade complex dietary carbohydrates [23-25]. A recent study conducted in five European centres reported a decrease in the abundance of *Bifidobacterium*, Enterobacteriaceae and certain species of *Clostridium*, whereas the abundance of members belonging to the *Clostridium coccoides *and *Clostridium leptum *groups increased after weaning [26]. Variations in dietary habits between Singapore and Indonesia may explain the differences in rates of colonization of these bacterial groups between Singapore and Indonesian subjects and therefore the slopes of the curves with age for *Bifidobacterium, Clostridium leptum *and *Bacteroides *(Figure 2).

A low relative abundance of the *Bacteroides-Prevotella *group was observed throughout all time points up till the age of 12 month (mean 7.31%). Our previous publication based on 16S rRNA pyrosequencing reported similar proportion of *Bacteroides *(8.90%) in healthy infants at 12 months [5] and substantiates the findings in this current study. On the contrary in adult the Bacteroidetes co-inhabits with the Firmicutes and both phyla dominate the bacterial community of the human gut microbiome [16,27,28]. The structure of the infant gut microbiome is dynamic and evolves over the first years of life toward an adult-like microbiota [29-31].

Besides monitoring for the temporal succession of stool microbiota, we further evaluate if demographic and lifestyle differences in the two studied geographical locations (Singapore, SG and Indonesia, IN) would influence the abundance of specific bacterial groups. A study conducted across Europe showed that the geographic origin had an impact on the composition of the gut microbiota [10], and it remains unknown if the structure of the microbiota is influenced to the same extent in Asia. In this study, both SG and IN differ in its extent of development and urbanization, and we observed a higher relative abundance of *Bifidobacterium *in the SG cohort compared to IN. This might be a common feature of urban populations, as it has also been reported previously for Northern European countries such as Stockholm to have a higher abundance of *Bifidobacterium *in infants stool microbiota as compared to those sampled in the Spanish province of Granada [10].

In addition, the two geographical locations in this study differ significantly in various aspects, for instance in mode of delivery, feeding history, occurrence of antibiotics consumption and sibling number. Interestingly, these factors studied have also been associated with the development of allergic diseases [32-35]. It has been postulated that the influence of these factors have on atopic disease may at least be in part through the effects on profile of gut microbiota. When we examined the effects of demographic and lifestyle factors, we found that the mode of delivery had the largest effect on stool microbiota of infants. These observations are supported by previous studies, where higher numbers of bacterial members belonging to the genus *Bifidobacterium *[36,37], *Bacteroides *and *Atopobium *group were observed for vaginal delivered infants compared to caesarean delivered infants [8,10]. Compared to caesarean delivered infants, the general notion is that infants who are vaginally delivered are exposed to their mothers' vaginal microbiota, in particular the *Lactobacillus *spp. [38], and therefore would have a higher incidence of bacterial transmission to their gut. However, in contrast to previous report which detected a higher abundance of *Lactobacillus *spp. in vaginally delivered infants [39], we detected a lower abundance of *Lactobacilli-Enterococci *group in our studied cohort. This discrepancy may be due to the specificities of different oligonucleotide primers/probes used to target the *Lactobacillus-Enterococci *group. Alternatively, the close adherence of *Lactobacillus *spp. to mucosal layers might hinder its transmission to the infants while the other vaginal microbiota gets transmitted to the infant [40]. Future validations on a larger cohort of vaginally delivered infants residing in SG and IN will be needed to verify the associated low abundance of *Lactobacillus*.

Our study also showed that vaginal delivered infants had a significantly higher number of terminal restriction fragments (T-RFs) and microbial richness at 12 months of age. Previous studies had reported that the diversity of stool microbiota increased over time [41]. We postulate that the higher abundance of beneficial bacteria such as *Bifidobacterium *associated with vaginal delivery may promote the diversity of overall gut microbiota as the infant ages.

Our findings also suggest that antibiotics consumption and sibling number are potential factors that influence the bacterial composition of the human fecal microbiota. For example, the consumption of postnatal antibiotic exposure resulted in a higher relative abundance of members of the *Clostridium leptum *group at one year of age. Previous studies have also found that postnatal antibiotic intake were associated with decreased numbers of *Bifidobacterium *and *Bacteroides *[11,42], further suggesting that antibiotics consumption can perturb the structure of the commensal microbiota. A higher abundance of *Bifidobacterium *was observed to be associated with the presence of older siblings [11]. Furthermore, we noted a corresponding decrease in the abundance of Enterobacteriaceae with the number of siblings. Interestingly, Lewis and colleagues have previously reported a decrease in the incidence of allergy with the number of siblings [34], while our past studies have found higher abundance of *Bifidobacterium *spp. and decreased abundance of Enterobacteriaceae in healthy infants compared to infants with eczema [5,6]. It remains to be further established if these multitude of factors: the sibship size and abundance of *Bifidobacterium *spp. and Enterobacteriaceae are intricately linked with the development of allergy and its related disorders. Besides demographic and lifestyle characteristics, the genetic make-up of the host has been proposed to be an important contributing factor in shaping the composition of the gut microbiota. Ambiguous findings were reported for twin studies on the influence of host genetics on gut microbiota [27,43], whereas absence, mutation or variation of certain single host genes were shown to affect the composition of gut microbiota [44].

FISH-FC approach showed a phylogenetic gap ranging from 22.89% to 37.40% of total bacteria for the four time points. A similar bacterial coverage was reported by Fallani *et al *using the same method, where the sum of bacterial cells detected were 72.7% ± 24.5% [10] and 74.3% ± 18.9% [45] with a panel of 10 non-overlapping probes. We acknowledge that the molecular techniques applied in this study do not permit a thorough description of the bacterial population inhabiting the human colon. Future studies would aim to utilize deep sequencing of the 16S rRNA genes so as to delve in depth the bacterial communities populating the human microbiome [46,47]. Their greater depths of sampling offer the opportunity to explore within the phylogenetic gap and beyond, therefore allowing high-resolution association studies involving the bacterial populations of the human microbiome as "quantitative traits".

## Conclusions

In conclusion, we have shown that variations in term of relative abundance in infant fecal microbiota are discernable for bacterial groups between two Asian populations of different geographical locations. The differences in the stool microbiota were partly explained by certain lifestyle and clinical factors. These features may confound studies relating to the association of stool microbiota and the predisposition to disease, and should be an important confounder to take note for comparative studies that enrol large population cohort across different geographical origins.

## Methods

### Subject recruitment and study design

The SG at risk of atopy cohort (n = 42) is a subgroup selected from the placebo arm (n = 112) of a randomized double-blind placebo controlled clinical trial on the administration of probiotics supplemented cow's milk-based infant formula for 6 months on the prevention of eczema and allergic diseases. The placebo group of the study received the same cow's milk-based infant formula without probiotics. This study was conducted at National University of Hospital, Singapore (ClinicalTrials.gov Identifier: NCT00318695) [48]. The Indonesia at risk of atopy cohort (n = 32) was selected from a birth cohort study (n = 66) recruited from expectant mothers who visited Gadjah Mada University Hospital, Yogyakarta. The inclusion criteria for both cohorts were 1) first-degree relative with a history of allergic disorder as confirmed by a doctor's diagnosis of asthma, allergic rhinitis, or eczema and a positive skin prick test to any of a panel of common dust mite allergens, which are the most important inhalant allergens in our atopic population [49]; 2) gestational age above 35 wk and birth weight above 2 kg; 3) absence of major congenital malformations or major illness at birth; 4) deemed to be in good health based on medical history and physical examination; and 5) the family assessed to be able to complete the trial. Consecutive cases with availability of stool sample with 3 or 4 time points for FISH-FC were selected from both SG and IN cohorts. The sample size was calculated based on study by Sepp et al [50], which reported a higher prevalence of Lactobacillus at 12 months of age in Estonian infants (63%) compared with Swedish infants (38%). We therefore anticipated the difference to be approximately 25% with a power of 90% and a two-sided test size of 5%, 49 subjects were required in each group. No probiotics and prebiotics consumption was reported for SG cohort during the early infancy. Four infants within the IN cohort were partially fed with milk formula that contained prebiotics. Written informed consent for participation in the study was obtained from the parents/guardians of all infants. The study was approved by the National University Hospital's ethics review committee (Ref Code: B/00/322).

### Stool sampling

Stool samples were collected on day 3, and at 1, 3, and 12 month after birth based on collection and processing produce as described previously [51]. Stool samples were collected into sterile plastic vials by parents, stored in the freezer at -20°C and delivered to the laboratory within 20 hours. The samples were kept cool on a dry-ice pack during transport and, immediately upon arrival at the laboratory, diluted with 0.85% sodium chloride solution (saline) to give a 0.1 g/ml homogenate. After preparation of the homogenate, samples were fixed in 4% paraformaldehyde (PFA), and stored in TN (10 mM Tris-HCl [pH 8], 150 mM NaCl) buffer at -80°C for later FISH-FC and DNA extraction respectively. Stool samples stored in PFA and TN buffer from Indonesia were shipped on dry-ice pack to single location (laboratory in National University of Singapore) for analysis.

### DNA extraction and T-RFLP Analysis

Bacterial DNA extractions from stool samples were carried out as described previously [51] using 0.3 g of 0.1 mm zirconia/silica beads (Biospec, Inc) and a mini bead-beater (Biospec, Inc). In brief, the aqueous supernatant containing DNA will be subsequently subjected to two phenol-chloroform (1:2) extractions and precipitated with 1 ml of ethanol and 50 μl of 3 M sodium acetate. Finally, DNA will be dissolved in 25 μl of sterile TE Buffer (pH 8.0) and stored at -20°C until analysis.

For T-RFLP analysis, 16S rRNA genes were amplified using primers 47F (5'- Cy5 -GCY TAA YAC ATG CAA GT -3') and 1492R (5'-GGY TAC CTT GTT ACG ACT T-3'). PCR reaction mix comprises of 50 ng of DNA template, 0.2 μM (each) of forward and reverse primers, 0.2 mM dNTP, 1.5 U of Ex Taq DNA polymerase (Takara Bio Inc., Japan) and the volume added up to 50 μl with molecular biological grade water. The PCR conditions were as follow: 3 mins at 95°C, 20 cycles (30 seconds at 95°C, 45 seconds at 50°C, 1 min at 72°C), and 5 mins at 72°C. After PCR amplification, mung bean digestion (New England Biolabs [NEB], MA) was carried out to digest single-stranded overhangs. PCR products were purified with QIAquick PCR purification kit (Qiagen, Germany) prior to individual enzymatic digestion by AluI, MspI and RsaI (NEB, MA). Terminal restricted fragments (T-RFs) were analyzed after capillary electrophoresis on CEQ8000 genetic analyzer (Beckman Coulter, CA) [52].

### Fluorescence *in situ *hybridization combined with flow cytometry (FISH-FC)

FISH-FC was performed as described previously [53]. A panel of seven bacterial phylogenetic probes was used as described previously by [51]. These probes were selected to target the *Eubacterium rectale-Clostridium coccoides *group (Erec 482), *Clostridium leptum *subgroup (Clep 866 and the corresponding competitor probes), *Bacteroides-Prevotella *group (Bac 303), *Bifidobacterium *genus (Bif 164), *Atopobium *group (Ato 291), *Lactobacilli-Enterococci *group (Lab 158) and Enterobacteriaceae family (Enter 1432). Eubacterial probe EUB338 was used as positive control, while NON 338 probe was used as negative control. Samples were analyzed with FACS Calibur flow cytometer (Becton-Dickinson, USA). A total of 100,000 cells were acquired for analysis per sample and bacterial concentrations adjusted to lower than 3,000 events/s. Subsequent analyses were conducted using the Cell Quest Software (Becton Dickinson, USA). True-positive counts were determined by subtracting double-labelled bacteria with background level evaluated with NON 338 probe. The relative abundance of each bacterial group was expressed as percentage of total EUB338 labelled bacterial cells.

### Statistical analysis

All statistical analyses were carried out using SPSS v.18 (SPSS: IBM, Chicago III, USA). Linear mixed model was used to evaluate the demographic effect and time effect (i.e. 4 time points) while adjusting for other confounders. The relative abundance of bacterial groups was used as dependent variable in the model. Three variance-covariance structures (compound symmetry, 1st order autoregressive and unstructured) were used for linear mixed model, and the selection of covariance structure was based on Akaike's Information Criterion (AIC) and Schwarz's Bayesian Criterion (BIC). Linear regression analyse was used to analyze the effects of the postnatal antibiotic consumption on the relative abundance of bacterial groups, with adjustment for confounding factors at single time point. Shannon and Simpson Index were calculated from relative intensity of T-RF as described previously [7], and were used as dependent variable in the model of linear regression to investigate the effects of confounding factors. Confounding factors used for adjustment were based on the all demographics factors being studied in this study and standardized for all statistical analysis (i.e, Linear mixed model and linear regression), those factors were location, mode of delivery, weaning age, sibling number, total breastfeeding up to 6 month, eczema and prenatal antibiotics. Statistical significance was set at p < 0.05. All statistical significance tests and confidence intervals were two-sided.

## Authors' contributions

GCY and KKC performed the experiments, data analysis and statistical analysis. GCY drafted the manuscript. PYH and BWL helped to revise the manuscript. CL helped in experimental techniques for FISH-FC. YDZ and DL participated in collation of clinical data and helped in statistical analysis. MA, LPCS and BWL conceived the study. DC, S, YS, MA, LPCS, KYC and BWL participated in study design and helped in coordination of sample and data collection. All authors read and approved the final manuscript.

## Supplementary Material

Additional file 1 **Univariate analysis of relative abundance of seven predominant bacterial groups**. Univariate analysis of relative abundance of seven predominant bacterial groups were performed for location, mode of delivery, total breastfeeding up to 6 month, eczema, prenatal antibiotics and postnatal antibiotics. Statistical significance were bold formatted (p value < 0.05). Click here for file
